# Fungal hepatic abscess formation postlaparoscopic cholecystectomy

**DOI:** 10.1093/jscr/rjae802

**Published:** 2024-12-21

**Authors:** Dana AlNuaimi, Ghufran Saeed, Shareefa Abdulghaffar, Reem AlKetbi, Essa M Aleassa, Numan Cem Balci

**Affiliations:** Department of Health, Abu Dhabi, United Arab Emirates; Sheikh Khalifa Medical City, Abu Dhabi, United Arab Emirates; Dubai Health, Dubai, United Arab Emirates; Dubai Health, Dubai, United Arab Emirates; Cleveland Clinic Abu Dhabi, Abu Dhabi, United Arab Emirates; Khalifa University, Abu Dhabi, United Arab Emirates; Cleveland Clinic Abu Dhabi, Abu Dhabi, United Arab Emirates; Cleveland Clinic Lerner College of Medicine, Cleveland, OH, United States

**Keywords:** laparoscopic cholecystectomy, fungal abscess, cholelithiasis, computed tomography, magnetic resonance imaging

## Abstract

Laparoscopic cholecystectomy is the preferred method for treating acute cholecystitis. Although the incidence of postoperative infections in laparoscopic cholecystectomy is low, serious postoperative surgical site infections are still reported. Hepatic abscesses, particularly fungal, can occur post-cholecystectomy leading to significant mortality and morbidity. We report a case of a 58-year-old female who underwent laparoscopic cholecystectomy and subsequently developed fever, jaundice, and right upper quadrant pain. Laboratory results showed deranged liver function tests with raised inflammatory markers. Radiographic investigations, including CT and MRI, revealed an irregular hilar lesion with periportal changes suggestive of an abscess with portal vein thrombosis. Histopathological examination of the biopsy obtained from the hilar lesion showed a fungal hepatic infection, and particularly conidiobolomycosis. To our best knowledge, this is the first case that reports this fungal infection as a complication of laparoscopic cholecystectomy. The patient was managed with a combination of intravenous antibiotics and antifungals, which yielded mild improvement. Unfortunately, the patient decided to leave the hospital against medical advice, limiting the information on the disease course.

## Introduction

Laparoscopic cholecystectomy has the advantage of better cosmesis, shorter length of hospital stay, and faster return of function in comparison to open cholecystectomy [1]. Although surgical and postoperative morbidity are low post-laparoscopic cholecystectomy, surgical site infections are still reported in the literature [2, 3]. The range of surgical site infection spans from superficial surgical site infections, to abdominal abscesses, and organ infections that vary in severity. In this case report, we report a case of fungal liver abscess post-laparoscopic cholecystectomy.

## Case report

This is a 53-year-old female patient who presented to the emergency department complaining of right upper quadrant abdominal pain, fever, and icterus of a few days duration.

She had undergone a laparoscopic cholecystectomy ~12 days earlier in another healthcare facility, after which the patient experienced right upper quadrant pain with repeated bouts of vomiting. She had no other significant medical or surgical history. On physical examination, the patient was febrile and jaundiced. The abdominal examination revealed healing laparoscopic cholecystectomy scars. The abdomen was tender over the right upper quadrant and epigastric areas.

Laboratory blood investigations showed elevated C-reactive protein level (209 mg/l), white blood cell count (19.3^3^ U/l), and absolute neutrophil and eosinophil counts. Patient was anemic with hemoglobin of 8.8 g/dl. Liver enzymes including gamma-glutamyl transferase (202 U/l), aspartate transferase, and alanine transferase (56 U/l) were elevated. Total bilirubin (10.2 mg/dl) and alkaline phosphatase (654 U/l) were extremely high. CA 19-9 was also high (146 U/ml) while AFP tumor marker was within normal limits.

**Figure 1:**
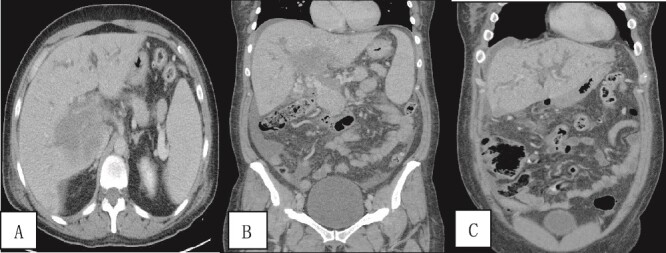
Contrast-enhanced CT scan of the abdomen and pelvis in (A) axial and (B, C) coronal sections obtained at the porto-venous phase showing a hypo-vascular geographic area with complex fluid density at the hilum of the liver with intrahepatic biliary dilatation. Non-opacification of the main portal vein with early cavernous transformation was noted as well as mild splenomegaly and ascites.

A contrast-enhanced CT scan of the abdomen and pelvis was done and revealed a dilated non-opacified main portal vein and superior mesenteric vein suggestive of thrombosis with periportal vascular congestion as well as a hypo-vascular geographic area with complex fluid density at the hilum of the liver and gallbladder fossa with dilated right and left hepatic biliary ducts. It also showed mild splenomegaly, colonic diverticulosis, and mild ascites mainly in the right iliac fossa. The splenic vein appeared normal, and there were no imaging features of bowel ischemia (Fig. 1).

**Figure 2:**
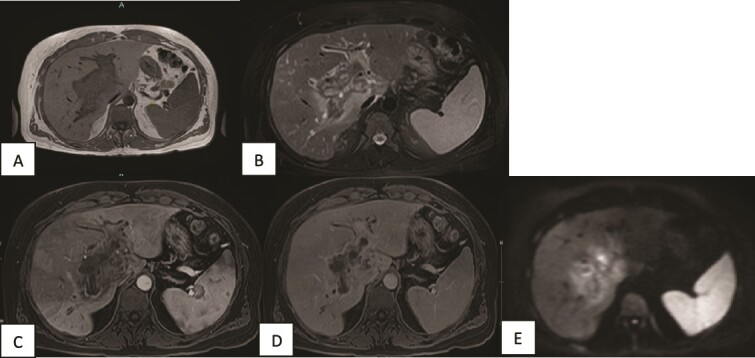
MRI of the liver (A, B) Axial T1WI and T2WI fat-saturated images revealing an isointense central rim with hypointense periportal edema on T1-weighted images and a hypointense rim along the portal tracts with periportal edema on T2-weighted images. (C, D) Axial T1WI postcontrast images obtained at the arterial and porto-venous phases showing mild peripheral enhancement with non-enhancement of the portal vein. (E) DWI showing minimal diffusion restriction at the hilum.

A contrast-enhanced MRI of the liver was done and showed periportal infiltrative soft tissue changes at the hilum of the liver with irregularly enhancing margins on contrast-enhanced T1-weighted images. There was also minimal diffusion restriction on diffusion-weighted images and which was confined to the periportal tracts involving the main portal and intrahepatic right and left periportal spaces. The presence of portal vein thrombosis was also confirmed (Fig. 2).

**Figure 3:**
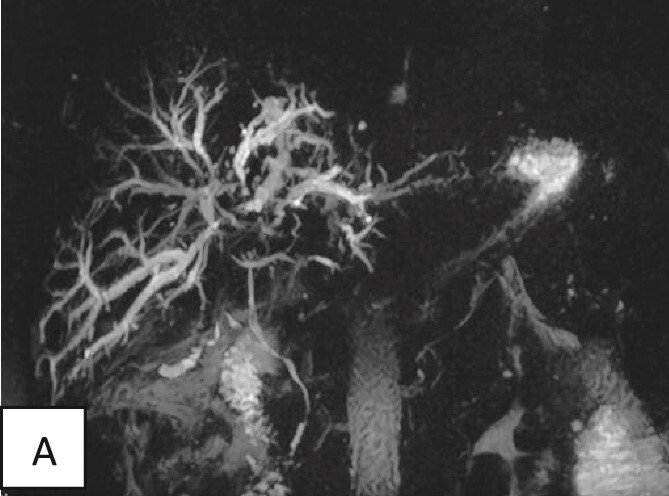
Magnetic resonance cholangiopancreatography of the liver showing dilated intrahepatic biliary radicals. Normal insertion and caliber of both cystic and common bile duct with no filling defects or irregularity to suggest impacted stones or peripheral strictures.

The magnetic resonance cholangiopancreatography showed intrahepatic biliary radical dilatation with periportal edematous changes. A small fluid collection in the surgical bed tracking along the subhepatic space was seen showing no post-contrast enhancement. A tortuous cystic duct measuring ~3–4 cm in length inserting into the proximal segment of the common bile duct was also noted. Nevertheless, the common bile duct was normal in diameter (3 mm) with no filling defects (Fig. 3).

Interpretation of the imaging and laboratory findings favored an infectious-inflammatory process with a collection at the hilum rather than malignant, i.e., perihilar cholangiocarcinoma with portal vein invasion.

The patient was admitted, and anticoagulation therapy was initiated. She was also started on intravenous antibiotics: meropenem and metronidazole. The patient’s bilirubin levels slightly decreased then increased again in the following days.

The patient’s condition did not improve with the anticoagulants and antibiotics, as she continued to be febrile and complain of right upper quadrant pain and did not tolerate food orally. Blood culture showed no growth after 5 days. The subsequent bilirubin levels remained high.

An endoscopic retrograde cholangiopancreatography was performed, which showed a stricture at the hilum and left hepatic system. The left was stented during the procedure while the right could not be stented due to technical difficulties. Later on, she underwent a percutaneous transhepatic cholangiography (PTC), the right hepatic system was drained and a biopsy was taken.

A pigtail catheter drain was placed in the right iliac fossa for drainage of ascites with analysis of the fluid suggestive of bacterial peritonitis. Blood culture was repeated, showing *E. coli* bacterial growth.

**Figure 4:**
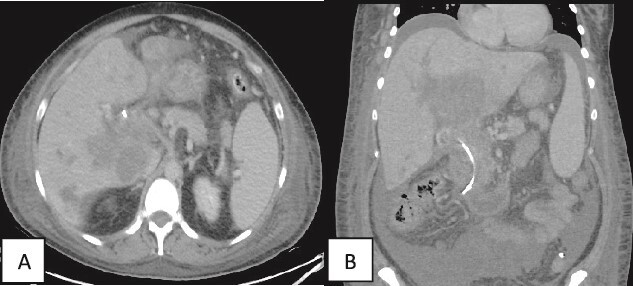
(A, B) Contrast enhanced CT scan of the abdomen and pelvis in axial and coronal sections in porto-venous phase status post-stenting showing that the hypo-vascular hypodense areas progressed compared to the initial CT with involvement of the liver parenchyma and hepatic hilum. Associated intrahepatic biliary ductal dilation and evidence of increased ascites was also noted. A pig-tail catheter tip is seen in the left lower quadrant.

Contrast enhanced CT scan of the abdomen and pelvis was repeated post-PTC and showed multiple intrahepatic fluid collections suggestive of abscess formation and an increase in ascites (Fig. 4).

**Figure 5:**
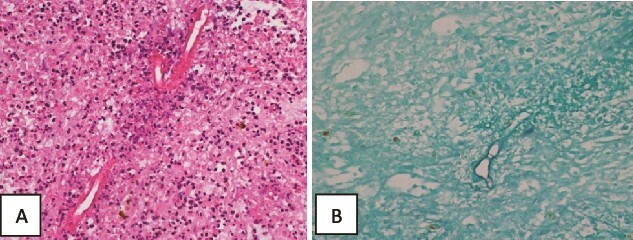
Histopathology of the liver core biopsy. (A) Inflamed necrotic/infarcted liver parenchymal tissue almost totally replaced by severe diffuse eosinophilic inflammatory infiltrates with Splendore–Hoeppli structures, Charcot Leyden crystals, hemorrhage, edema and fibrosis consistent with a hepatic abscess. Hematoxylin and eosin section ×400. (B) GMS special stain reveals large broad variably sized mostly aseptate thin-walled fungal hyphae with branching morphologically consistent with zygomycotic fungal hyphae ×400.

Histopathology of the liver biopsy showed granulomas and multinucleated giant cells with foci of necrotic tissue. It also showed parasite-like or fungal spore-like structures with foci of Splendore–Hoeppli phenomenon-related changes. Massive hepatic necrosis with foci of viable hepatocytic parenchyma showing acute hepatitis, micro abscesses, focal microvesicular steatosis, cholestasis, and heavy pigmentation suggestive of obstructive ascending cholangitis-related changes was also noted. No evidence of malignancy was seen (Fig. 5).

These findings were consistent with zygomycotic fungal hyphae and in particular conidiobolomycosis (entomophthoromycosis) fungal infection of the liver with bacterial peritonitis and ascites.

The patient was started on antifungal medication in addition to the IV antibiotics treatment with continuation of anticoagulation, and she showed mild clinical improvement. Nevertheless, the patient decided to leave against medical advice and return to her country of origin.

## Discussion

Liver abscesses, particularly fungal, can be a serious potential complication post-cholecystectomy leading to significant mortality and morbidity even in immunocompetent patients [4]. Fungal infections are more evident in critically ill and immunocompromised patients, with Candida being the predominant pathogen, while Aspergillus infections are more common in immunocompetent patients [5].

As fungal liver abscess formation has the potential to arise in immunocompetent patients with no risk factors, it requires a high index of clinical suspicion. Likewise, candida can also be found in the bile of patients with biliary obstruction [4, 6].

Entomophthoromycosis is a fungal infection, which is more common in tropical and subtropical areas, occurring otherwise in both healthy immunocompetent and immunocompromised patients. It tends to grow in fatty media in the subcutaneous and mucocutaneous tissue and is thought to be developed by spore's inhalation causing sinus or skin disease [7, 8]. It is often non-life-threatening but can result in extended morbidity if early diagnosis and proper treatment were not established. Moreover, some invasive forms may cause fatal dissemination, especially in immunocompromised patients or patients with systemic illness [9, 10].

Patients with disseminated disease can present with masses in the liver and gallbladder fossa as seen in our case.

Two types of entomophthoromycosis are recognized: basidiobolomycosis (subcutaneous zygomycosis) and conidiobolomycosis (rhinofacial zygomycosis). Conidiobolomycosis is unable to penetrate a healthy intact skin, but they can penetrate a traumatic wound by secreting lipolytic and proteolytic enzymes and can remain in the host subcutaneous tissues for years as chronic granulomas [10].

The gold standard approach for diagnosis is usually made through histopathological examination of a biopsy specimen obtained from the edge of the lesion [10]. Wet mount studies are utilized in obtaining the definite diagnosis and in cases with negative cultures due to potential damage of the tissue samples during processing. PCR studies can be done on a fresh frozen section [10].

Diagnostic imaging with CT or MRI is important during image-guided specimen collection, in evaluating the extent of the disease and monitoring the treatment response [10, 11].

Entomophthoromycosis usually requires prolonged combined antifungal treatment, possibly with aggressive surgical debridement, given that the mortality rate can reach up to 50% despite early aggressive therapy [11, 12].

To our best knowledge, this is the first case to report this fungal infection as a complication of a recent laparoscopic cholecystectomy. The infection might have spread from skin at the surgical site to the gallbladder bed and liver. Another possibility is hematogenous spread.

Although rare, particularly in noncirrhotic liver, portal vein thrombosis is a recognized complication postlaparoscopic cholecystectomy in the literature [13, 14]. Risk factors can include increased body mass index, prothrombotic conditions, use of oral contraceptives, pancreatitis, blunt abdominal trauma, and intra-abdominal infection or malignancy [15].

The fungal infection the patient had in our case report is likely to be the underlying cause of developing portal vein thrombosis given that no other underlying cause could be identified.

In conclusion, fungal infections and hepatic abscess formation after a recent laparoscopic cholecystectomy are extremely rare. Medical imaging plays an important role in image-guided sample collection, assessing the extent of the disease and monitoring the response to treatment. Nevertheless, histopathological examination of a biopsy obtained from the lesion remains the gold standard for establishing the diagnosis.

Informed consent for the case to be published, including images, case history, and data was obtained from the patient for this case report.
